# Noninvasive Photochemical Sealing for Achilles Tendon Rupture by Combination of Upconversion Nanoparticles and Photochemical Tissue Bonding Technology

**DOI:** 10.1155/2020/1753152

**Published:** 2020-05-18

**Authors:** Yiming Zhu, Aiguo Xie, Ming Li, Chihao Zhang, Tao Ni

**Affiliations:** ^1^Department of General Surgery, Shanghai Ninth People's Hospital, School of Medicine, Shanghai Jiao Tong University, Shanghai 200011, China; ^2^Department of Plastic and Reconstructive Surgery, Shanghai Ninth People's Hospital, School of Medicine, Shanghai Jiao Tong University, Shanghai 200011, China

## Abstract

Photochemical tissue bonding (PTB), based on photosensitizer rose bengal (RB) and green light, has been regarded as an effective alternative to surgical suture and has been reported to provide benefits for Achilles tendon repair. Limited to the poor penetration of green light, secondary damage still exists while applying PTB for closed Achilles tendon rupture. This study is aimed at exploring the effects of noninvasive photochemical sealing on Achilles tendon rupture by the combination of PTB and upconversion nanoparticles (UCNPs). The rare-earth UCNPs of NaYF_4_ : Yb/Er (Y : Yb : Er = 78 : 20 : 2) were fabricated and then loaded into Chitosan/*β*-GP hydrogel containing RB to prepare UCNPs@RB/Chitosan/*β*-GP hydrogel. The properties of UCNPs and UCNP/Chitosan/*β*-GP hydrogel were characterized by TEM, SEM, DLS, and FTIR analysis. The effects of UCNP and PTB combination were evaluated in an Achilles tendon rupture rat model using histological analysis. Bioluminescence imaging of ROS was performed to explore the potential mechanism. UCNPs had a uniform shape with a diameter of 29.7 ± 2.6 nm. The UCNPs@RB/Chitosan/*β*-GP hydrogel could upconvert the near-infrared light into green light. The results of histological assessment showed that compared with traditional suture repair, the rats injected with UCNPs@RB/Chitosan/*β*-GP hydrogel followed by irradiating with near-infrared light and the rats treated with RB solution followed by irradiating with green light had better effects on Achilles tendon repair. The benefits might be related to the generation of ROS in the PTB process. These findings indicated that the combination of PTB and UCNPs@RB/Chitosan/*β*-GP hydrogel could be used as a noninvasive photochemical sealing for Achilles tendon rupture.

## 1. Introduction

Achilles tendon rupture is widely reported, and its incidence increases every year. In Denmark, the incidence of acute Achilles tendon rupture increased from 26.95 per 10,000 persons per year in 1994 to 31.17 per 10,000 persons per year in 2013 [1]. Operative and nonoperative methods are broadly used for the treatment of Achilles tendon rupture. Compared with the nonoperative treatment, the operative treatment had a lower risk of rerupture [2–5]. However, operative therapy increased the risk of many complications, including infection, adhesions, sural nerve injury, deep vein thrombosis, and disturbed skin sensibility [2–5].

Photochemical tissue bonding (PTB), which was first developed by the researchers from Massachusetts General Hospital, Harvard Medical School, is a novel suture-less technique for tissue repair and is regarded as an effective alternative to surgical suture [6]. The mechanisms of PTB depend on two components: photosensitizer rose bengal (RB) and green light. RB could absorb the photons of green laser and thus generate ROS reacting with amino acids in structural proteins, in which covalent binding between proteins, including collagen crosslinking, will be promoted [7]. Chan et al. has reported that PTB promotes tendon repair at early stages in a full thickness Achilles tendon transection rat model [8]. In our previous study, we found that a combination of PTB and electrospun silk provided benefits for tendon healing in an Achilles tendon transection rabbit model [9]. Recently, PTB has been reported to bond human amniotic membrane with the surface of the flexor digitorum profundus tendon and thus promote hand tendon repair better than surgical suture [10]. All these reports suggest that PTB can be a potential surgical technology for ruptured tendon repair. However, limited to the poor penetration of green light while applying PTB technology in closed tendon rupture injury, an incision to the skin to expose the ruptured tendon site is still needed, which will cause secondary damage. Whether there is a noninvasive sealing approach for the repair of ruptured tendon repair attracts our attention.

Upconversion nanoparticles (UCNPs), which can convert radiation with a longer wavelength (e.g., infrared or near-infrared light) to radiation with a shorter wavelength (e.g., visible or ultraviolet light) by a multiphoton mechanism, have attracted considerable attention for their application in medicine [11]. Based on the properties of UCNPs, Han et al. had reported the effective effect of the combination of PTB with UCNPs for the repair of dissected skin tissues [12]. Thus, if we convert the penetrating near-infrared light to green light by UCNPs, secondary damage will be avoided in the application of PTB in tendon repair. As a natural component of crab or shrimp shells, Chitosan is a biologically compatible and degradable polymer [13]. The aqueous formulations of Chitosan containing *β*-glycerophosphate (Chitosan/*β*-GP) can be easily administered by injection, and when injected in vivo, Chitosan/*β*-GP undergoes solution-gel transition at a temperature close to 37°C [13, 14]. The resulting Chitosan/*β*-GP hydrogel possesses superior biocompatibility [14]. Thus, Chitosan/*β*-GP hydrogel was applied to load UCNPs.

Here, we prepared an injectable Chitosan/*β*-GP hydrogel to load RB and UCNPs, which could convert near-infrared light to green light. The resulting solutions were injected into the site of a transected Achilles tendon and then turned into hydrogel in situ. Then, near-infrared light irradiation was used at the transected site through the skin. The effects of UCNP and PTB combination on Achilles tendon repair in rats were evaluated by Tunel assay, hematoxylin-eosin (H&E) staining, and Masson's trichrome staining.

## 2. Materials and Methods

### 2.1. Preparation of UCNPs and Injectable UCNP/RB/Chitosan Hydrogel

La_2_O_3_, Yb_2_O_3_, Y_2_O_3_, and Er_2_O_3_ were completely dissolved in 10% HCl under heating conditions to form hexahydrate chloride. After drying in vacuum, the abovementioned hexahydrate chloride was dissolved in ultrapure water and ethanol and then n-hexane was added. A reflux reaction was performed at 80°C for 4 h. The mixture was divided into two layers. The lower layer was removed by a separation funnel. The upper layer was washed with water for several times followed by drying in vacuum to obtain a waxy substance. A rare-earth oleic acid precursor was obtained by dissolving the above waxy substance in oleic acid under nitrogen circumstance. Then, equal amounts of the rare-earth oleic acid precursor and sodium oleate were dissolved in oleic acid and stirred violently to form a uniform solution. The uniform solution was mixed with BmimBF_4_ followed by heating in a hydrothermal reactor for 24 h at 240°C. After cooling at room temperature, the UCNPs of NaYF_4_ : Yb/Er were generated at the junction of the oil phase and ionic liquid phase. Finally, the UCNPs were collected and washed alternately with cyclohexane and ethanol for several times. After drying at 240°C for 12 h in vacuum, the prepared UCNPs were stored for subsequent experiments.

For the preparation of UCNPs@RB/Chitosan/*β*-GP hydrogel, Chitosan chloride, beta-glycerin sodium phosphate (*β*-GP), hydroxyethyl cellulose (HEC), and UCNPs were dissolved and dispersed in ultrapure water at 4°C (pH = 8). Then, after the addition of RB, the aqueous solution of UCNPs@RB/Chitosan/*β*-GP was obtained. The above solutions turned into gel in the mold at 37°C.

### 2.2. Characterization of UCNPs and UCNP/Chitosan/*β*-GP Hydrogel

The morphological structure of UCNPs was studied by JEM-2100 transmission electron microscopy (TEM; JEOL, Tokyo, Japan). The particle size of UCNPs was detected by dynamic light-scattering assay with a Malvern Zetasizer NanoZS instrument. The morphological structure of UCNP/Chitosan/*β*-GP hydrogel was studied by JSM-7001F scanning electron microscopy (SEM; JEOL, Tokyo, Japan). Fourier transform infrared spectroscopy (FTIR) analysis was performed to analyze the intermolecular interactions among various components of UCNP/Chitosan/*β*-GP hydrogel with a scan range from 4000 to 650 cm^−1^ on a Nicolet 5700 spectrophotometer (Thermo Fisher Scientific, Waltham, MA, USA).

### 2.3. Cell Culture

The rat tenocytes were maintained in DMEM containing 10% fetal bovine serum and 1% penicillin-streptomycin and cultured at 37°C and 5% CO_2_.

### 2.4. Cytotoxicity Assay

The cytotoxicity of UCNP/RB/Chitosan/*β*-GP hydrogel on tenocytes was determined by CCK-8 assay. Tenocytes were cultured on the surface of UCNP/RB/Chitosan hydrogel. After having been incubated with Chitosan/*β*-GP, RB/Chitosan/*β*-GP, and UCNP/RB/Chitosan/*β*-GP hydrogel for 1, 4, and 7 days, the tenocytes were planted into a 96-well plate.

### 2.5. Achilles Tendon Injury Rat Model

The experiments were approved by the Research Animal Care Subcommittee of Shanghai Ninth People's Hospital, School of Medicine, Shanghai Jiao Tong University. Male Sprague Dawley rats (220-250 g) were obtained from Shanghai Jiesijie Experimental Animal Co., Ltd. (Shanghai, China). All rats were divided into four groups: the normal group; the suture repair group (SR group); the RB+532 nm group; and the UCNPs@hydrogel+808 nm group.

The rats were anesthetized by intraperitoneal injection of 2% pentobarbital sodium (2.5 mL/kg weight). The Achilles tendon in the right hind limb was exposed and partly transected surgically. In the SR group, the transected sites were repaired with standard Kessler suture. In the RB+532 nm group, the transected site was treated with RB solution containing ROS Brite™ 700 (16004; AAT Bioquest, Sunnyvale, CA) and irradiated with 532 nm green light for 2 min for once. Then, the skin was sutured. In the UCNPs@hydrogel+808 nm group, the skin was firstly sutured after transection. Subsequently, UCNPs@RB/Chitosan/*β*-GP hydrogel containing ROS Brite™ 700 was injected into the transected site and irradiation with 808 nm near-infrared light was applied at 1, 4, and 7 days post operation. The rats without transection and irradiation were used as normal control.

### 2.6. Tunel Assay

The tissues (1.5 cm × 1.5 cm × 0.3 cm) were obtained from the ruptured Achilles tendon site and fixed in 10% formalin for 48 h. Then, the tissues were embedded in paraffin and cut into 4 ~ 7 *μ*m slices. The cell death in different groups was detected using the *In Situ* Cell Death Detection Kit (11684817910, Roche, Mannheim, Germany) per the manufacturer's instruction.

### 2.7. Histological Assessment

The aforementioned 4 ~ 7 *μ*m slices were used for H&E staining and Masson's trichrome staining. H&E staining was applied to assess the structure and arrangement of the fiber and cell density. Masson's trichrome staining was performed to evaluate collagen deposition at the transected site of the Achilles tendon.

### 2.8. Bioluminescence Imaging of ROS

All rats in the RB+532 nm group and UCNPs@RB-hydrogel+808 nm group were probed with ROS Brite™ 700. The bioluminescence imaging of ROS was performed on an *in vivo* imaging instrument (IVIS Lumina III; PerkinElmer, Waltham, MA, USA). After LED irradiation (680 nm wavelength), the 706 nm wavelength signals were acquired.

### 2.9. Statistical Analysis

The data was represented as mean ± standard deviation. Statistical analysis was performed using SPSS 22.0. Statistical difference among groups was analyzed using one-way analysis of variance followed by Tukey's test. *P* value < 0.05 was considered as significant difference.

## 3. Results

### 3.1. Characterization of UCNPs and UCNP/Chitosan Hydrogel

**Figure 1:**
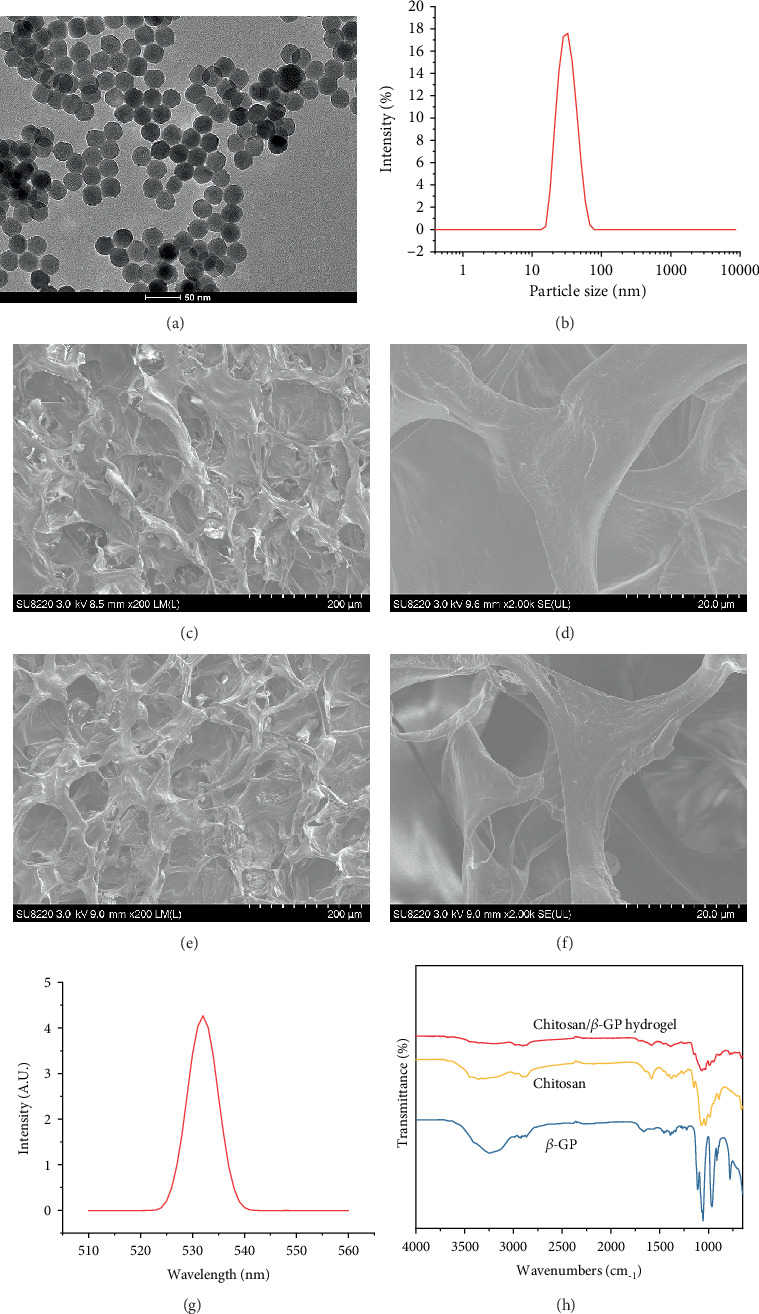
Characterization of UCNPs and UCNP/Chitosan/*β*-GP hydrogel. (a). TEM images of UCNPs. (b). Particle size of UCNPs. SEM images of Chitosan/*β*-GP hydrogel (c and d) and UCNP/Chitosan/*β*-GP hydrogel (e and f). (g). Photoluminescence intensity of UCNP/Chitosan/*β*-GP hydrogel under 808 nm near-infrared light excitation. (h). FTIR analysis of Chitosan, *β*-GP, and Chitosan/*β*-GP hydrogel.

As shown in Figures 1(a) and 1(b), the prepared UCNPs of NaYF_4_ : Yb/Er (Y : Yb : Er = 78 : 20 : 2) had a uniform shape with a particle size of 29.7 ± 2.6 nm. The addition of UCNPs did not affect the morphological structure of Chitosan/*β*-GP hydrogel (Figures 1(c) and 1(d)). By adjusting the content of different components in the UCNPs, the UCNPs could convert the near-infrared light (808 nm) into green light (532 nm) (Figure 1(e)). Additionally, the FTIR spectra of Chitosan, *β*-GP, and Chitosan/*β*-GP are shown in Figure 1(f).

**Figure 2:**
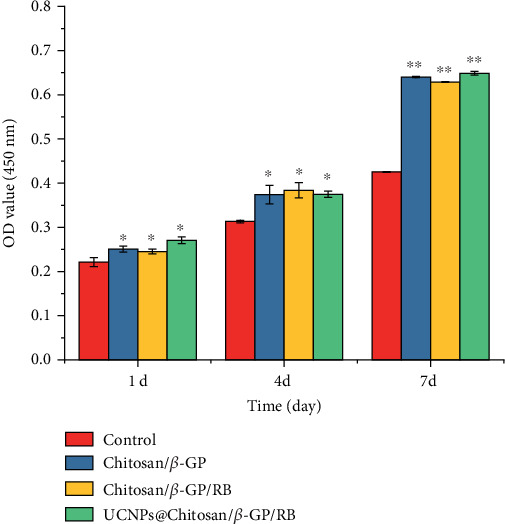
Cell viability analysis of human tenocytes incubated with different components in UCNP/RB/Chitosan/*β*-GP hydrogel. ^∗^*p* < 0.05 and ^∗∗^*p* < 0.01, compared with control.

The cytotoxicity of Chitosan/*β*-GP, RB/Chitosan/*β*-GP, and UCNPs@RB/Chitosan/*β*-GP hydrogels in the tenocytes was assessed with the CCK-8 assay. After 1, 4, and 7 days of incubation with Chitosan/*β*-GP, RB/Chitosan/*β*-GP and UCNPs@RB/Chitosan/*β*-GP hydrogels, the cell viability of the tenocytes was even significantly higher than that in the control group (Figure 2).

### 3.2. Observation of Cell Apoptosis

**Figure 3:**
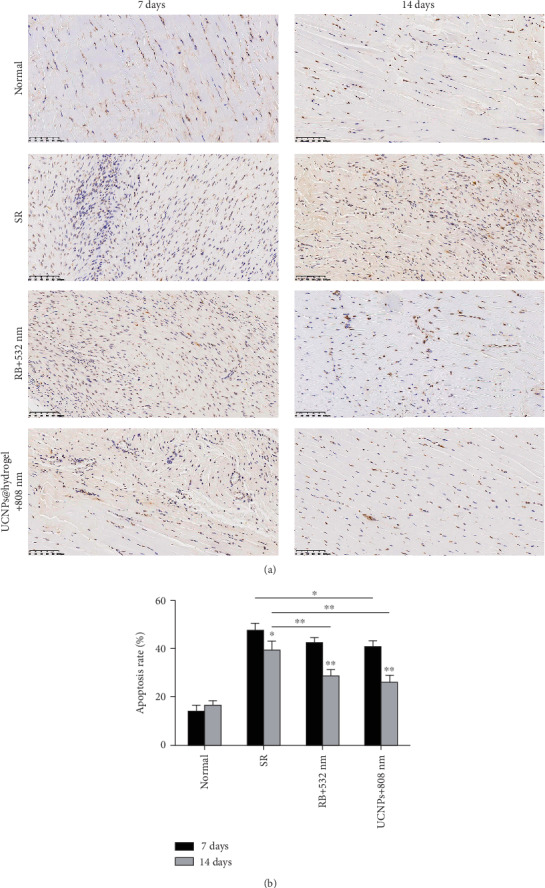
The cell apoptosis for the Achilles tendon site in different groups at 7 days and 14 days post operation. (a) Representative images of cell apoptosis by Tunel assay. (b) The apoptosis rate analysis. ^∗^*p* < 0.05 and ^∗∗^*p* < 0.01.

In the normal Achilles tendon, there are a small number of cells (Figure 3(a)). The number of live cells obviously increased in the three experimental groups (the SR, RB+532 nm, and UCNPs@hydrogel+808 nm groups) at 7 days post operation, while the cells were visibly reduced at 14 days post operation, especially in the RB+532 nm group and the UCNPs@RB-hydrogel+808 nm group. Compared with the normal group, the three experimental groups had a significantly increased cell apoptosis rate at 7 days and 14 days post operation (Figure 3(b)). Compared with 7 days post operation, the cell apoptosis rate in all the three experimental groups decreased significantly at 14 days post operation. In the early stage of tendon repair, the cell apoptosis rate obviously increased, while, subsequently, the cell apoptosis rate obviously decreased. Notably, the apoptosis rate in the RB+532 nm and UCNPs@hydrogel+808 nm groups are much lower than that in the SR group at 14 days post operation, suggesting better effects on tendon repair.

### 3.3. Histological Analysis of Achilles Tendon Healing

**Figure 4:**
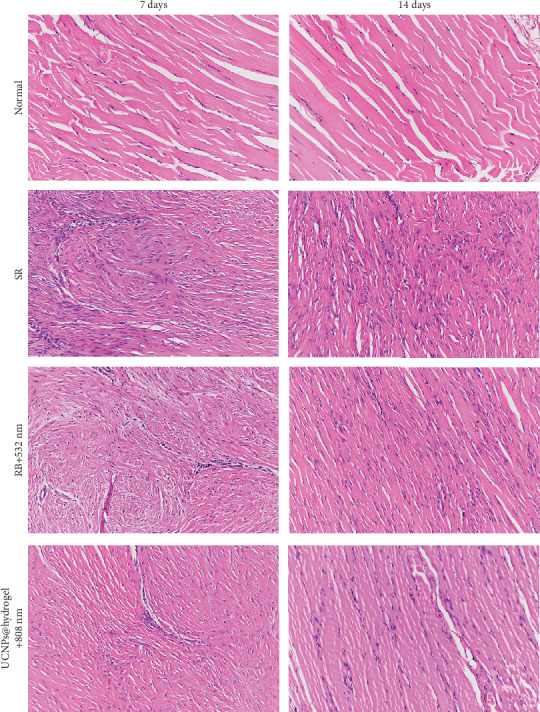
Representative images of hematoxylin and eosin staining for the Achilles tendon site in different groups at 7 days and 14 days post operation.

To evaluate the effect of different treatments on Achilles tendon repair, histological analysis was observed using H&E staining and Masson's trichrome staining. H&E staining showed that the normal Achilles tendon presented an integrated and continuous phenotype (Figure 4). At 7 days post operation, the Achilles tendon in the SR, RB+532 nm, and UCNPs@RB-hydrogel+808 nm groups appeared disordered. At 14 days post operation, the repaired Achilles tendon appeared much more integrated and continuous in the RB+532 nm and UCNPs@RB-hydrogel+808 nm groups than in the SR group, especially in the UCNPs@RB-hydrogel+808 nm group.

**Figure 5:**
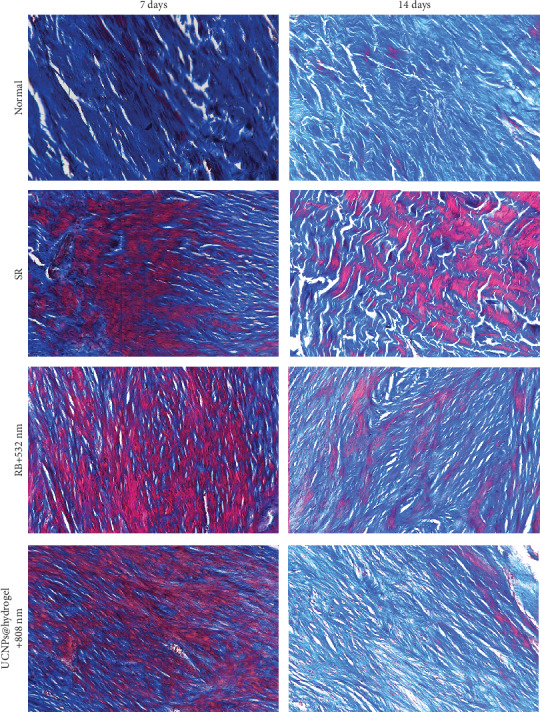
Representative images of Masson's trichrome staining for the Achilles tendon site in different groups at 7 days and 14 days post operation.

Masson's trichrome staining showed that there were dense collagen fibers in the normal Achilles tendon, while the collagen fibers were much fewer in the SR, RB+532 nm, and UCNPs@RB-hydrogel+808 nm groups at 7 days post operation (Figure 5). At 14 days post operation, the content of collagen fibers in the RB+532 nm and UCNPs@RB-hydrogel+808 nm groups was obviously higher than that in the SR group and was quite similar to that of the normal group. Moreover, compared with the RB+532 nm group, the collagen fibers were a little denser in UCNPs@RB-hydrogel+808 nm group.

### 3.4. Bioluminescence Imaging of ROS

**Figure 6:**
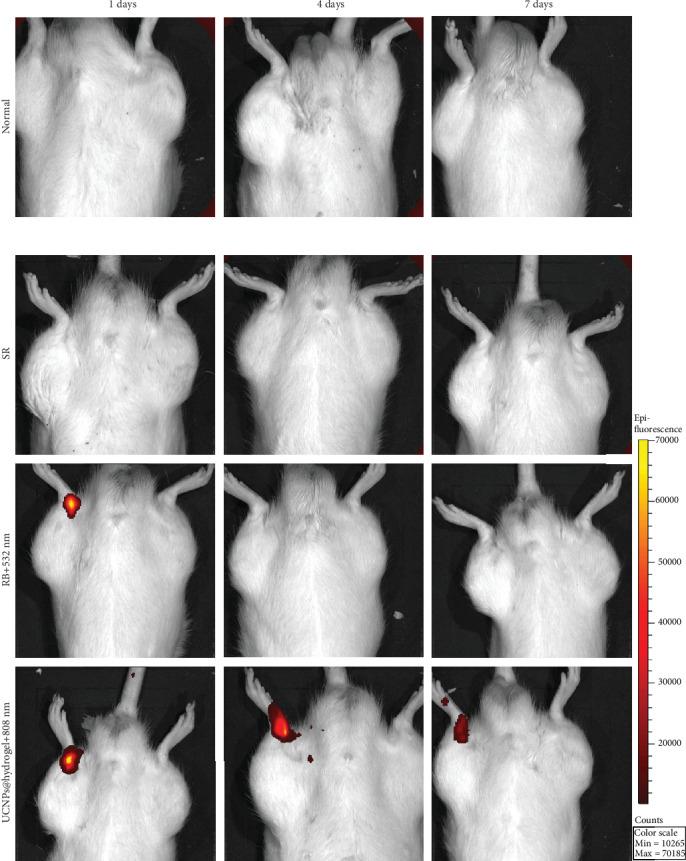
Bioluminescence imaging of ROS at the ruptured Achilles tendon site in different groups at 1, 4, and 7 days post operation.

As shown in Figure 6, there was no bioluminescence in the normal and SR groups. Bioluminescence was observed at 1 day post operation in the RB+532 nm group and at 1, 4, and 7 days post operation in the UCNPs@RB-hydrogel+808 nm group. Moreover, the bioluminescence intensity in the UCNPs@RB-hydrogel+808 nm group decreased markedly over time.

## 4. Discussion

Photochemical tissue bonding (PTB), based on photosensitizer rose bengal (RB) and green light, is an emerging suture-less biomedical application technology for tissue repair. PTB has been reported to be applied in numerous tissues, such as skin [6, 15–17], intestine [18], stomach [19], cornea [20, 21], nerves [22, 23], and blood vessels [24, 25]. For tendon repair, PTB has been reported to promote tendon repair at early stages in a full thickness Achilles tendon transection rat model [8]. Our previous study showed that the combination of PTB and electrospun silk provided benefits for tendon healing in an Achilles tendon transection rabbit model [9]. PTB also bonded human amniotic membrane with the surface of the flexor digitorum profundus tendon and thus promoted hand tendon repair better than surgical suture [10]. However, limited to the poor penetration of green light, while applying PTB in closed tendon rupture, the ruptured Achilles tendon has to be exposed by skin incision, which will cause secondary damage. This study explores the effects of noninvasive photochemical sealing on Achilles tendon rupture by a combination of PTB and UCNPs.

According to a previous study, compared with nontreated control, PTB treatment, which applied RB and green light, is beneficial for the repair of a transected Achilles tendon in rat [8]. In this study, evidenced by the results of Tunel assay, H&E staining, and Masson's trichrome staining, we demonstrated that in comparison with the traditional suture repair, the noninvasive photochemical sealing using modified PTB, which applied UCNPs@RB/Chitosan/*β*-GP hydrogel and near-infrared light instead of green light, had better effects on Achilles tendon repair, while in comparison with the traditional PTB treatment, this noninvasive photochemical sealing had similar, even better effects on the repair of a transected Achilles tendon in the rat model.

Here, we also found that bioluminescence, which suggested the generation of ROS, was observed at 1 day post operation in the RB+532 nm group and at 1, 3, and 7 days post operation in the UCNPs@RB-hydrogel+808 nm group. Moreover, the bioluminescence intensity for ROS in the UCNPs@RB-hydrogel+808 nm group decreased markedly over time. Our previous study showed that PTB treatment could significantly induce the generation of ROS and could promote cell proliferation and migration in tenocytes [26]. Thus, the effects of PTB or modified PTB with UCNPs might be attributed to the generation of ROS. Moreover, suitably more frequent generation of ROS in the UCNPs@RB-hydrogel+808 nm group led to even better effects than that for only once in the RB+532 nm group.

In conclusion, injectable hydrogels containing RB and UCNPs (UCNPs@RB/Chitosan/*β*-GP hydrogel), which could convert near-infrared light to green light, were prepared and were not toxic to tenocytes. Based on the peculiarity of UCNPs, PTB technology was modified and exerted its role on the site of a transected Achilles tendon without incising skin. Therefore, the combination of PTB and UCNPs@RB/Chitosan/*β*-GP hydrogel could be used as a noninvasive photochemical sealing for Achilles tendon rupture.

## Data Availability

The data used to support the findings of this study are included within the article.
